# Diagnostic limitations in advanced stage peripheral arterial disease in a cadaveric study comparing photon-counting and energy-integrating CT detectors

**DOI:** 10.1038/s41598-025-91239-x

**Published:** 2025-02-26

**Authors:** Viktor Hartung, Henner Huflage, Anne Marie Augustin, Sven Lichthardt, Dominik Peter, Florian Kleefeldt, Süleyman Ergün, Thorsten Alexander Bley, Jan-Peter Grunz, Philipp Gruschwitz

**Affiliations:** 1 Department of Diagnostic and Interventional Radiology, University Hospital Würzburg, Oberdürrbacher Str. 6, 97080 Würzburg, Germany; 2 Department of General, Visceral, Transplant, Vascular and Pediatric Surgery, Center of Operative Medicine, University Hospital Würzburg, Würzburg, Germany; 3 Institute of Anatomy and Cell Biology, University of Würzburg, Würzburg, Germany; 4 Department of Interventional and Diagnostic Radiology, Bayreuth Hospital, Bayreuth, Germany; 5 Department of Radiology, University of Wisconsin–Madison, Madison, WI USA

**Keywords:** Photon-counting CT, Image quality, Peripheral arterial disease, Experimental models of disease, Preclinical research, Cardiovascular diseases

## Abstract

To evaluate the limits of 1st-generation dual-source photon-counting detector CT (PCD-CT) and 3rd-generation dual-source energy-integrating-detector (EID-CT) regarding imaging of advanced stage peripheral arterial disease (ASPAD) of the femoral runoff. One human cadaver with ASPAD of the superficial femoral arteries was surgically prepared to establish continuous extracorporeal perfusion of the right upper leg. In addition to one stent already in place, three more stents were deployed in positions with severe calcification and stenosis to create thirteen different scenarios of ASPAD. CT angiographies with different radiation dose (CTDI_vol_ 10, 5, 3 mGy) and matching convolution kernels were performed with PCD-CT and EID-CT. In-stent lumen visibility, signal-to-noise ratio (SNR), and luminal attenuation were assessed quantitatively. Results were compared using analyses of variance with a PCD-CT maximum dose and resolution scan (96 mGy, BV89) serving as standard of reference. Highest and lowest stent lumen visibility was observed with PCD-CT BV76 (97 ± 2%) and EID BV40 (77 ± 5%), respectively. Severe stent underexpansion in conjunction with heavy calcification resulted in the worst lumen visibility. PCD-CT displayed superior dose efficiency, yielding comparable SNR at 3 mGy to EID-CT at 10 mGy (p = 0.27). Luminal attenuation was higher for PCD-CT regardless of dose and reconstruction settings (max. 369 ± 19 HU, BV76, 5 mGy vs. 329 ± 12 HU for EID, BV59, 5 mGy; p < 0.001). PCD-CT realises substantially higher image quality than EID-CT, thereby enhancing assessment of the femoral vasculature in ASPAD. Furthermore, this indicates substantial radiation dose and contrast agent volume saving potential. Both scanners show limitations in very low luminal diameters.

## Introduction

Peripheral arterial disease (PAD) remains a highly prevalent but frequently underdiagnosed condition, posing a considerable impact on public health^1^. Computed tomography (CT) is the cornerstone for evaluating vascular status and plays a pivotal role in the strategic planning for vascular interventions in lower extremity PAD but also represents the main source of radiation exposure in patient care^2,3^.

Endovascular therapy provides various advantages, including lower complication rates, reduced hospitalization duration as well as decreased rates of amputation and mortality, in comparison to traditional surgical interventions^4^. The option of repeating procedures to maintain long-term patency further underscores the importance of accurately determining patient eligibility for endovascular treatments. CT angiography (CTA) is widely accepted as the standard diagnostic modality for evaluating PAD, particularly in the femoral runoff^5–7^. However, the efficacy of CTA in advanced PAD stages is often compromised by severe arterial calcification and the presence of stents, which can obscure the vascular lumen and this has noteworthy impact on the decision for endovascular therapy versus bypass surgery, particularly in complex cases.

Energy-integrating detector CT (EID-CT) scanners are known to be limited in precisely delineating small vessels, significant calcifications, and stent lumens^8,9^. In contrast, the advent of photon-counting detector CT (PCD-CT) technology promises enhanced dose efficiency^10–13^ and substantial improvements in image quality^14–19^. This advancement is particularly evident in the imaging of the femoral runoff, as demonstrated in both phantom studies ^20,21^ and patient cohorts^22,23^.

Extending on previous investigations using extracorporeally-perfused human cadaveric models^24^, this study aims to leverage this innovative in-vitro model to explore the capabilities and limitations of PCD-CT and contemporary EID-CT in diagnostic assessment of advanced-stage PAD with the leading question, whether the results from previous work with disease-free cadavers can be confirmed. Furthermore, this study aims to allow a direct comparison between detector generations.

## Methods

### Cadaveric specimens

The anatomical institute of our university provided a fresh-frozen body donor. The cadaver was chosen intentionally with signs of ASPAD. One stent was already implanted in the right superficial femoral artery during the donor’s lifetime.

### Ethics declaration

The study protocol was approved by the institutional review board of the University Hospital Würzburg (protocol number: 20220413 01). All experiments complied with applicable laws and regulations. The body donor had provided informed consent in writing to the use of the cadaver for study and research purposes during the lifetime. While personal information was not disclosed following applicable regulations, the donor appeared to be of normal weight.

### Extracorporeal perfusion

The cadaveric perfusion model has been used successfully in other studies^20,21^ and its technicalities have been elucidated elsewhere^24^. In short, a board-certified vascular surgeon established vascular access to the common femoral and popliteal artery in the right leg. Continuous perfusion was established via conventional introducer sheaths. A peristaltic pump connected to the side ports of the sheaths and a reservoir of perfusion fluid (mixture of glucose and Ringer’s solution warmed to 37 °C) ensured continuous perfusion of the superficial femoral artery. An additional working sheath was placed parallel in the common femoral artery to allow placement of stents during continuous perfusion. The superficial femoral artery was chosen as target vasculature owing to its clinical significance in PAD, surgical accessibility, simplicity of stent deployment, and minimal collateral perfusion.

### Stent placement

**Fig. 1:**
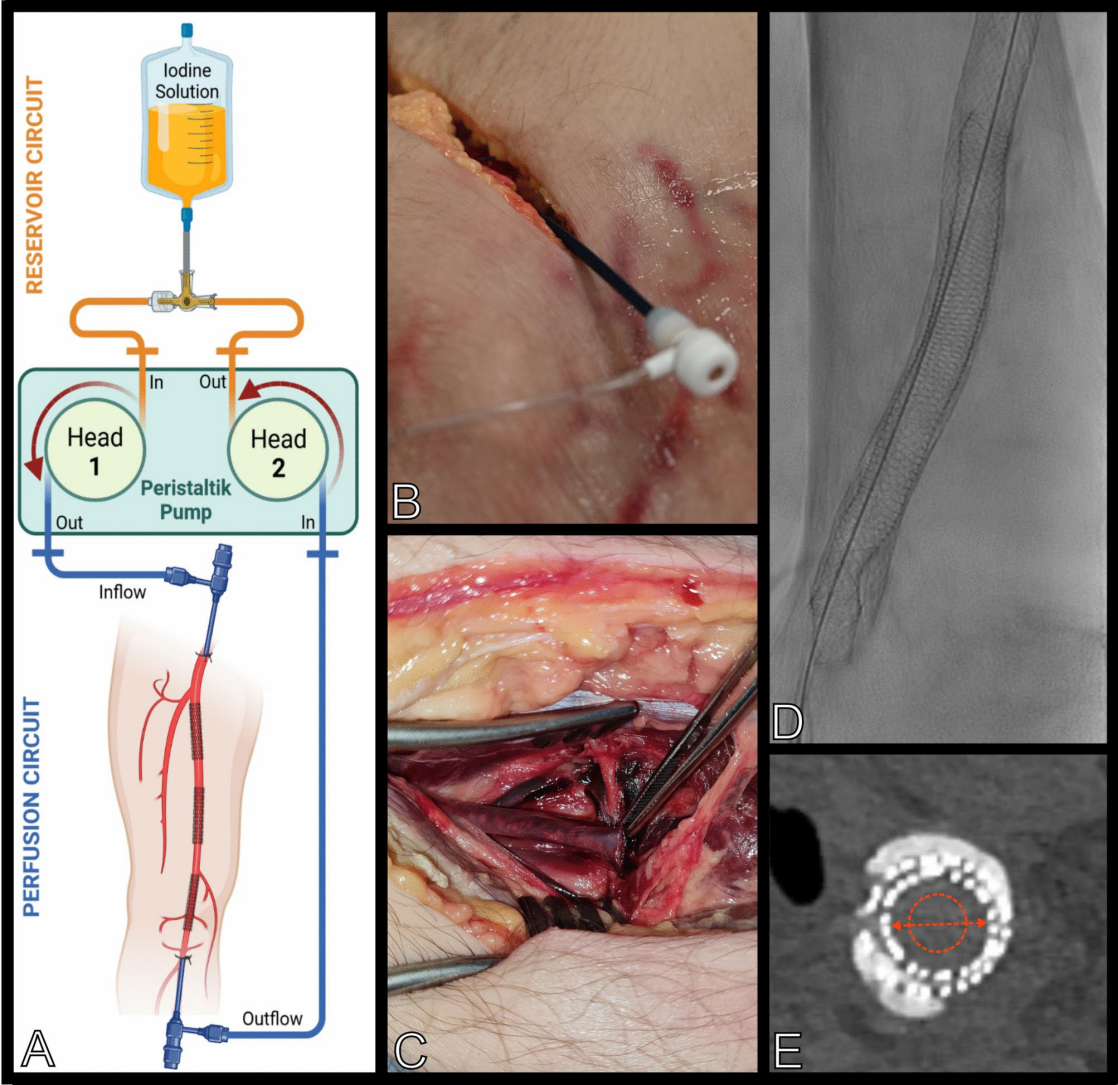
Perfusion model, stent deployment and measurements. (**A**) Schematic display of the cadaveric model with perfusion circuit (image created using biorender.com®; (**B**) surgical preparation of the groin with inflow sheath in place; (**C**) surgical preparation of the infragenicular popliteal outflow; (**D**) fluoroscopic image of a SUPERA stent (Abbott) placed inside a Wallstent (Boston Scientific). Notice the distal in-stent stenosis; (**E**) corresponding CT slice from the reference scan (UHR-PCD-CT, BV89, 96 mGy) with measurements of the stent lumen diameter and luminal attenuation indicated in red.

After preparation, the cadaver was transferred to a dedicated angiography suite. Conventional digital subtraction angiographies were performed under continuous extracorporeal perfusion with a pump flow rate of about 50 ml/min (Fig. 1). One stent was already implanted in the mid superficial femoral artery (Wallstent, 8 × 80 mm, Boston Scientific). Three other stent models were deployed in the same vessel by a board-certified radiologist with seven years of training in the field. One of them was intentionally oversized (Absolute Pro and SUPERA, Abbott Cardiovascular, Plymotuh, MN, USA). Self-expanding stent models with different mesh designs were utilized. Stent characteristics are listed in Table 1 and representative images of stent implantation are shown in Fig. 1. Perfusion with warmed solution allowed for complete expansion of the self-expanding stents verified by fluoroscopy control. Post-dilation was omitted intentionally.

**Table 1:** Characteristics of the utilized stents.

Manufacturer	Model	Nominal diameter (mm)	Length (mm)	Type	Material
Abbott	Supera	5.5	60	Self-expanding	Nitinol
Abbott	Absolute Pro	9	60	Self-expanding	Nitinol
Abbott	Absolute Pro	6	60	Self-expanding	Nitinol
Boston	Wallstent	8	80	Self-expanding	Nitinol

### Setup of PAD scenarios

**Fig. 2:**
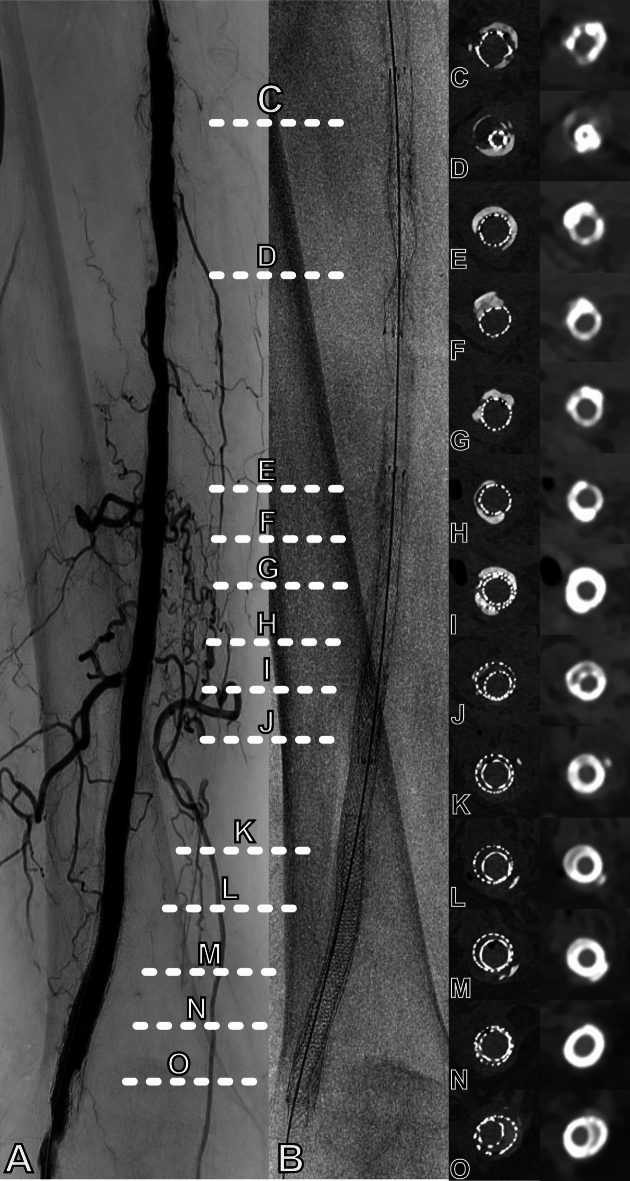
Different advanced stage peripheral arterial disease scenarios. (**A**) Digital subtraction angiography of the right upper leg after stent deployment with several stenoses; (**B**) corresponding fluoroscopy with depiction of stents and heavy calcifications; **C–O**: Axial slices of the evaluated scenarios with stent underexpansion (**C**), severe stent underexpansion (**D**), stent true-to-size with concurrent severe calcification (**E, G, H**), stent true-to-size with eccentric compression by severe calcification (**F**), stent-in-stent with inner stent true-to-size and severe calcification (**I**), stent-in-stent with underexpansion of the inner stent (**J–L, O**), and stent-in-stent with the inner stent true-to-size without relevant calcifications (**M, N**). Left column depicts the maximum-dose reference scan, while right column depicts images from standard-resolution PCD-CT (BV40) at 3 mGy. White dotted lines indicate the levels of measurements.

Several stent-related conditions in ASPAD were investigated: stent underexpansion, late incomplete stent apposition, stent collapse, and stent-in-stent situations. All these conditions usually occur in combination with differing degrees of stenosis and calcification, a scenario which was emulated in this experimental study. Stent underexpansion was simulated through two different approaches: First, stents were chosen true-to-size and released in regions with preexisting stenosis. Second, stents were oversized at about 50% of the reference vessel diameter to simulate higher stent strut densities owing to stent collapse. In addition, stents were nested into each other with or without vascular stenosis, underexpansion and calcification. Measurements were performed at 13 different locations to assess the simulated scenarios in presence of differing degrees of arterial calcification (Table 2). Corresponding digital subtraction angiography and CTA images are visualized in Fig. 2.

**Table 2:** Overview of the simulated scenarios existing in advanced stage peripheral arterial disease.

Position	Description	Stent model (outer)	Stent model (inner)	Stent diameter [mm]	Eff. Inner diameter [mm]
1	Stent underexpansion		Absolute Pro 9 × 60 mm	9	5.14
2	Stent underexpansion, severe calcification		Absolute Pro 9 × 60 mm	9	2.09
3	Stent true to size, severe calcification		Absolute Pro 9 × 60 mm	6	5.30
4	Stent true to size, eccentric compression, severe calcification		Absolute Pro 9 × 60 mm	6	5.02
5	Stent true to size, severe calcification		Absolute Pro 6 × 60 mm	6	4.86
6	Stent true to size, severe calcification		Absolute Pro 6 × 60 mm	6	5.17
7	Stent in stent, true to size, severe calcification	Wallstent 8 × 80 mm	Absolute Pro 6 × 60 mm	6	4.94
8	Stent in stent, underexpansion	Wallstent 8 × 80 mm	Absolute Pro 6 × 60 mm	6	4.20
9	Stent in stent, underexpansion	Wallstent 8 × 80 mm	SUPERA 5.5 × 60 mm	5.5	4.54
10	Stent in stent, underexpansion	Wallstent 8 × 80 mm	SUPERA 5.5 × 60 mm	5.5	4.66
11	Stent in stent, true to size	Wallstent 8 × 80 mm	SUPERA 5.5 × 60 mm	5.5	4.87
12	Stent in stent, true to size	Wallstent 8 × 80 mm	SUPERA 5.5 × 60 mm	5.5	4.98
13	Stent in stent, underexpansion	Wallstent 8 × 80 mm	SUPERA 5.5 × 60 mm	5.5	4.18

### Scan parameters

After successful stent deployment, CTA were performed with a mixture of the perfusion fluid and an iodinated contrast medium (Peritrast® 400 mg/ml, Dr. Franz Köhler Chemie GmbH, Bensheim, Germany) in a 30:1 dilution, corresponding to an iodine concentration of 12.9 mg/ml. This ratio has been determined in a preceding dilution series to attain a clinically acceptable luminal attenuation of 400 Hounsfield units (HU) with a 120 kVp PCD-CT high-dose scan (CTDI_vol_ 10 mGy).

All scan protocols and reconstructions were conducted resembling clinical routine as closely as possible. Dose levels were chosen based on national reference values detemining lower extremity CT angiographies at 7 mGy and the long-term average at the own institution at approximately 5 mGy, whereas 3 mGy and 10 mGy protocols would provide valuable information on the performance at reasonable dose level boundaries.

CTA scans were performed with a 1st-generation dual-source PCD-CT (Naeotom Alpha, Siemens Healthineers, Erlangen, Germany) and a 3rd-generation dual-source EID-CT (Somatom Force, Siemens Healthineers). An additional PCD-CT scan with maximum radiation dose was conducted in ultra-high resolution mode (UHR) with individual pixel readout and an effective tube current of 1200 mAs (96 mGy, max-dose) to serve as standard of reference. For comparison of both scanner models, radiation-dose matched scans were acquired using three different dose levels, defined as high dose (10 mGy), medium dose (5 mGy) and low dose (3 mGy). For comparability reasons, both scanners were operated with a fixed tube voltage of 120 kVp without automatic tube current modulation. PCD-CT imaging was performed in standard-resolution mode with 2 × 2 pixel binning (physical pixel size 0.275 × 0.322 mm^2^). The latest software versions at the time of data collection were used for both scanners (VA40—PCD-CT; VB10B—EID-CT). Scan parameters are summarized in the Table 3.

**Table 3:** Scan parameters and image reconstruction settings.

Scan parameters	EID-CT			PCD-CT			Ref. PCD-CT
Tube current (kVp)	120						
CTDI_vol_ (mGy)	3	5	10	3	5	10	96
Pitch	0.4						
Collimation (mm)	96 × 0.6			144 × 0.4			120 × 0.2
Z-Coverage (mm)	57.6			57.6			24

Image reconstruction	EID-CT			PCD-CT				Ref. PCD-CT
Field of view (mm^2^)	150 × 150							
Slice thickness, increment (mm)	1.0/1.0							
Convolution kernel	BV40	BV49	BV59	BV40	BV48	BV60	BV76	BV89
MTF (ρ_50_ in lp/cm)	3.95	5.62	8.32	3.95	5.4	8.79	16.47	26.78
Image matrix	512 × 512			512 × 512			768 × 768	1024 × 1024
Iterative reconstruction	ADMIRE level 3			QIR level 3				

### Image reconstruction

Raw data of both scanners were reconstructed with identical geometrical settings employing an image matrix of 512 × 512 pixels in a field of view of 150 × 150 mm^2^ and a slice thickness and increment of 1.0 mm each. Vascular convolution kernels with matching modulation transfer functions were applied for EID-CT (BV40 [ρ_50_ = 3.95 lp/cm], BV 49 [5.62 lp/cm], BV59 [8.32 lp/cm]) and PCD-CT (BV40 [3.95 lp/cm], BV48 [5.4 lp/cm], BV60 [8.79 lp/cm]). In addition, PCD-CT scans were reconstructed with ultra-sharp kernels (BV76 [16.47 lp/cm], BV89 [26.78 lp/cm] for the reference scan) not available for EID-CT. Comparable iterative reconstruction algorithms, which are also rouinely employed in clinical routine, were used (ADMIRE level 3 for EID-CT, QIR level 3 for PCD-CT, both Siemens Healthineers). To ensure maximum resolution yield depending on kernel capabilities, the image matrix was increased to 768 × 768 for BV76 and 1024 × 1024 for BV89 reconstructions.

### Objective image analysis

Figure 1E illustrates manual measurements performed by one board-certified radiologist with 10 years of experience in cardiovascular and interventional radiology in strictly axial reformations using the clinical picture archiving and communication system (Merlin, Phönix-PACS, Freiburg, Germany). Window settings were predefined to 1500/400 (window width/center).

### Stent lumen visibility

Addressing the eccentric deflection of the stent wall frequently encountered in ASPAD, stent lumen diameters were measured in matching orientations choosing the shortest diameter. To minimize quantitative errors, measurements were averaged from three adjoining slices, while beam hardening artifacts, stent strut interstices, and blooming were carefully avoided (Fig. 1E)^25^. UHR-PCD-CT max-dose images reconstructed with the ultra-sharp BV89 kernel and a 1024 × 1024 pixel matrix served as the quantitative reference standard. Stent lumen visibility was defined as the averaged stent diameter measured for each scanner-kernel combination divided by the reference scan measurement.

### Luminal attenuation

Luminal attenuation was analysed by manually placing intra-arterial regions of interest as large as possible within the stent lumen, while avoiding stent struts and artifacts (HU_stent_) (Fig. 1). Luminal attenuation was assessed in absolute HU values and as HU difference compared to unaltered vessel segments to define the influence of blooming, beam hardening, and photon starvation caused by stents and calcification.

### Signal-to-noise ratio

To assess image noise, a predefined region of interest of 75 mm^2^ was drawn in unaltered fat tissue close to the stent or vessel of interest. Noise was measured as the standard deviation of the fat tissue attenuation (SD_fat_) and not inside the stent lumen to ensure sufficient ROI size and avoid bias from artifacts introduced by calcification and stents. Based on manual measurements, signal-to-noise ratios (SNR) were calculated as $SNR = HU_{{stent}} /SD_{{fat}}$.

### Statistical analysis

Analyses were performed using dedicated software (Prism 10.3, Graphpad Software Inc., CA, USA). Statistical significance is indicated by p values < 0.05. Continuous variables are reported as mean ± standard deviation. Items were compared using one-way analyses of variance for repeated measures with the null hypothesis, that PCD-CT would perform superior to EID-CT. P values of pairwise post-hoc tests were corrected for multiple comparisons by applying the Šídák method.

## Results

### Application of the perfusion model in ASPAD

While the perfusion model could be established satisfactorily, leak tightness was impaired at the inflow and outflow introducer sites due to severe calcification, which resulted in substantial leakage of contrast material into the perivascular space and surrounding soft tissues. However, this did not impair CT angiographies or subsequent image analysis. Angiographic imaging and stent placement could also be performed as usual. The correlation of CTA and digital subtraction angiography allowed for accurate stent deployment to create the different ASPAD scenarios.

### Stent lumen visibility

**Fig. 3:**
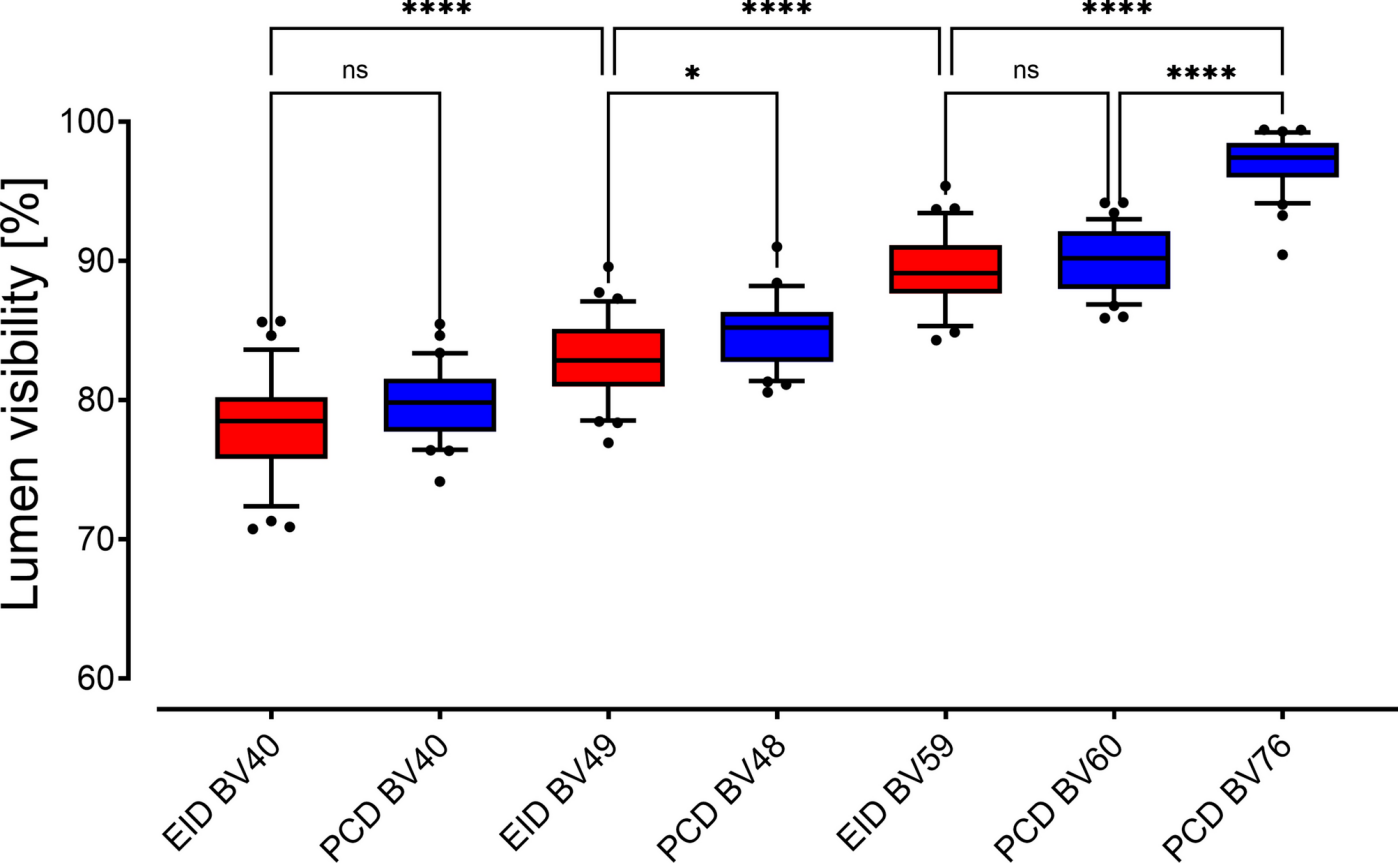
Boxplot diagram of lumen visibility. Lumen visibility in percent of the reference diameter. Measurements were pooled by scanner and kernel. Note. – EID-CT: red boxplots, PCD-CT: blue boxplots. Brackets refer to statistical analysis, whereby ns indicates p ≥ 0.05, * indicates p ≤ 0.05, ** indicates p ≤ 0.01, *** indicates p ≤ 0.001 and **** indicates p ≤ 0.0001.

Radiation dose had no significant influence on lumen visibility, regardless of the detector technology or reconstruction kernel (all p > 0.600, Supplemental Table 1). Consequently, for further analysis, data of all dose levels were pooled and sorted by kernel. PCD-CT and EID-CT achieved comparable lumen visibility for reconstruction kernels with comparable sharpness. PCD-CT images reconstructed with the ultra-sharp BV76 kernel displayed better stent lumen visibility than the best EID-CT result with the sharpest applicable kernel BV59 (97 ± 2% versus 88 ± 6%; p < 0.001; Fig. 3). Over all ASPAD scenarios, EID-CT reconstructed with BV40 resulted in the lowest average lumen visibility of 77 ± 5%. Among the simulated scenarios, severe stent underexpansion lead to the lowest stent lumen visibility of 66.7% (scenario 2, Table 2, for reference see Fig. 2D).

### Signal-to-noise ratio and resolution

Within arterial segments with severe stent underexpansion (maximum inner diameter of 2.09 mm, scenario 2, Table 2), considerable artifacts obscured the luminal attenuation. This fact was particularly evident when soft kernels were used. The luminal attenuation exceeded 655 HU for EID-CT BV40 and 785 HU for PCD-CT BV40; both measurements were considerably higher than in unaltered, un-stented vessel segments. As a result, we decided to exclude scenario 2 for statistical SNR comparisons.

**Fig. 4:**
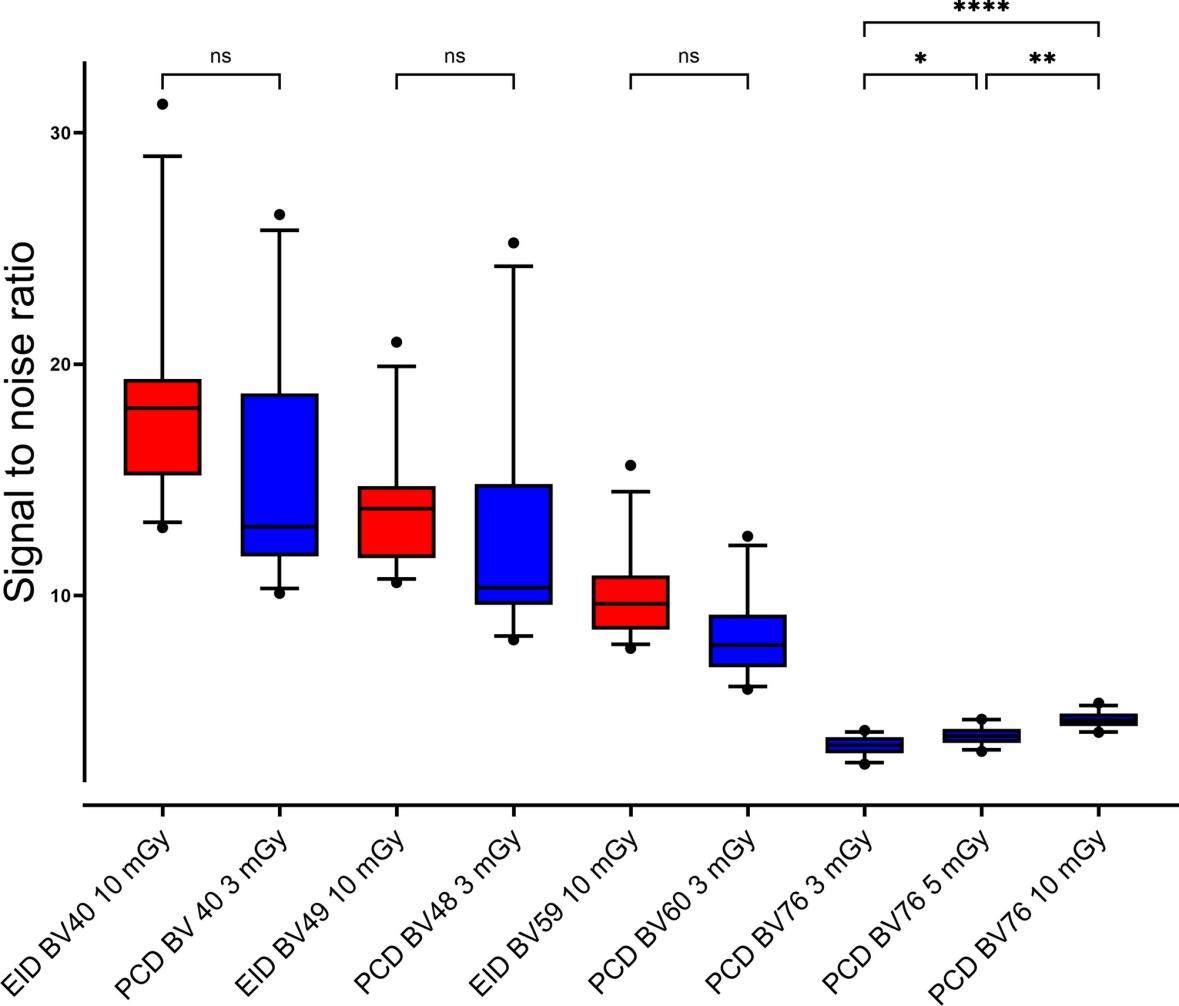
Boxplot diagram of signal-to-noise ratios. Low-dose PCD-CT scans deliver comparable SNR to high-dose EID-CT scans. On the right, SNR values of PCD-CT data reconstructed with the sharp BV76 kernel are depicted. Data from scenario 2 (i.e., severe stent underexpansion) was omitted due to extensive artifacts. Note. – EID-CT: red boxplots, PCD-CT: blue boxplots. Brackets refer to statistical analysis, whereby ns indicates p ≥ 0.05, * indicates p ≤ 0.05, ** indicates p ≤ 0.01, *** indicates p ≤ 0.001 and **** indicates p ≤ 0.0001.

PCD-CT (BV40) at 10 mGy achieved the highest SNR with 16.3 ± 7.2. SNR of PCD-CT (BV60) at 3 mGy was comparable to EID-CT (BV59) at 10 mGy (8.3 ± 2.0 versus 9.9 ± 2.1; p = 0.320). Quantitative results are summarized in Fig. 4. Only PCD-CT scans reconstructed with the ultra-sharp BV76 kernel showed a dose-dependent SNR increase from 3.5 ± 0.4 at 3 mGy over 3.9 ± 0.4 at 5 mGy (p = 0.024) to 4.6 ± 0.4 at 10 mGy (p = 0.007).

### Luminal attenuation

**Fig. 5:**
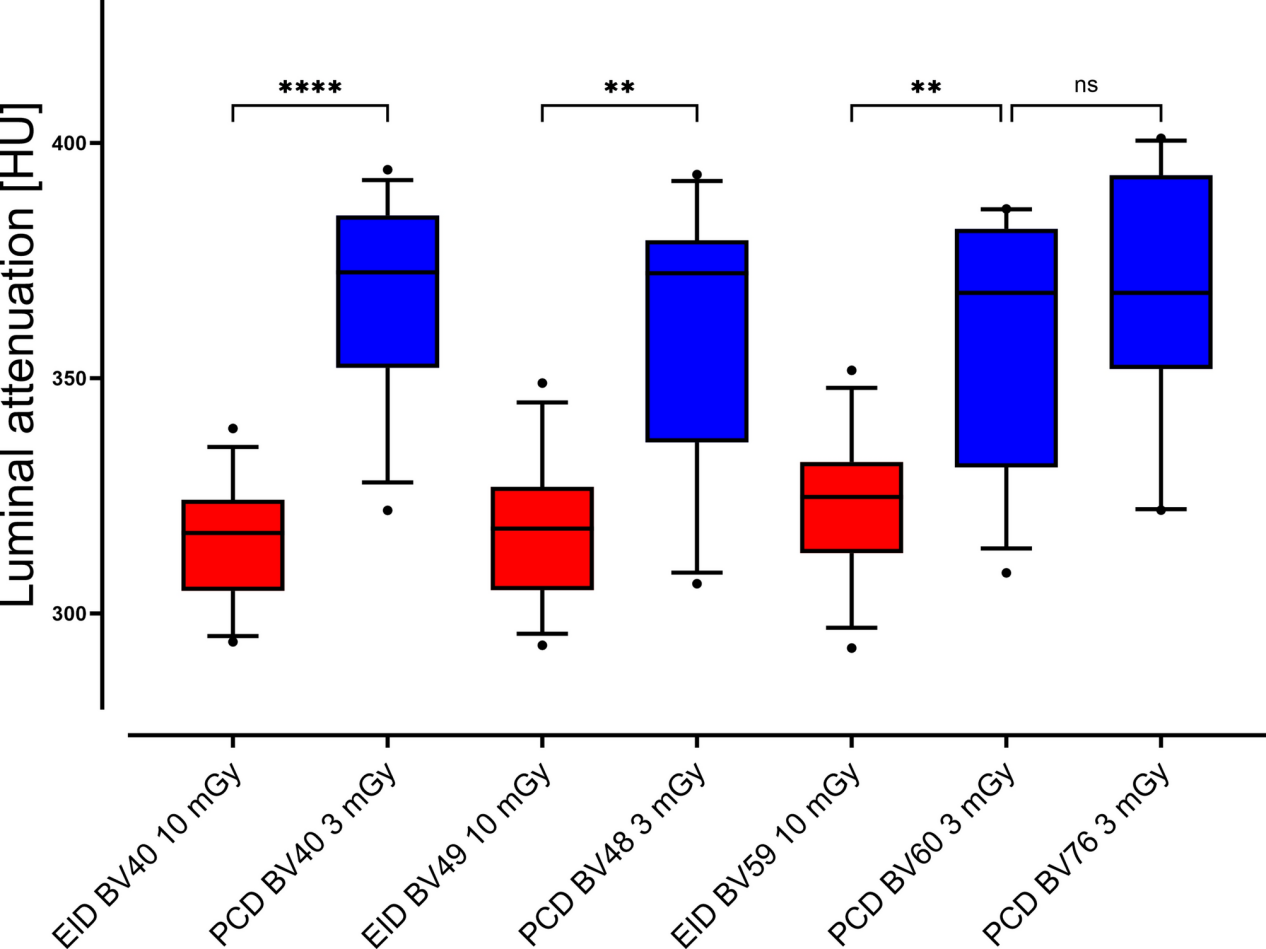
Boxplot diagram of luminal attenuation. Luminal attenuation in PCD-CT images is higher than in EID-CT images. Data from scenario 2 (i.e., severe stent underexpansion) was omitted due to extensive artifacts. Note. – EID-CT: red boxplots, PCD-CT: blue boxplots. Brackets refer to statistical analysis, whereby ns indicates p ≥ 0.05, * indicates p ≤ 0.05, ** indicates p ≤ 0.01, *** indicates p ≤ 0.001 and **** indicates p ≤ 0.0001.

PCD-CT achieved higher absolute luminal attenuation than EID-CT, regardless of ASPAD scenario, reconstruction kernel, or radiation dose (all p < 0.025). Highest attenuation values were measured in PCD-CT (BV60) at 10 mGy (360 ± 20 HU) and PCD-CT (BV76) at 5 mGy (369 ± 19 HU), while EID-CT (BV59) at 5 mGy did not exceed 329 ± 12 HU. Luminal attenuation values are summarized in Fig. 5.

Variation of luminal attenuation within stents and adjoining un-stented segments was insignificant in most scenarios and did not exceed 22.9 ± 16.2 HU (i.e., stent-in-stent with underexpansion of the inner stent, scenario 13, Table 2). An exception was observed in scenario 2 (i.e., severe stent underexpansion with residual luminal diameter of 2.09 mm) with considerable variation for both scanners up to + 270.5 HU for EID-CT (BV40) at 3 mGy and + 294.2 HU for PCD-CT (BV40) at 3 mGy.

## Discussion

This study compared the performances of 1st-generation photon-counting detector CT and 3rd-generation energy-integrating detector CT in an extracorporeally perfused human cadaver model with advanced stage peripheral arterial disease. We were able to simulate a broad range of disease scenarios including several settings rarely encountered in clinical practice. The realism of this model significantly surpasses previous work in healthy subjects^21^ and consequently, the results are more likely to be directly transferable into clinical routine despite their phantom model character. Photon-counting detector CT performed significantly better in several key aspects of image quality, such as stent lumen visibility and luminal contrast attenuation. By including a body donor with advanced stage peripheral arterial disease, our investigation alleviates the legitimate criticism with of less refined models not demonstrating actual stenosis and calcification.

Our results demonstrate that a small residual luminal diameter obscures the lumen more than extensive calcification, high stent strut density, or multiple stent layers. Stent lumen visibility was above 60% of the true diameter for both PCD-CT and EID-CT, regardless of scan and reconstruction settings. This should facilitate the detection of clinically relevant stenosis in even the most challenging cases of ASPAD if the artery diameter is above 4 mm. For vessel diameters of less than 2 mm and considerable contraction of stent struts, both CT scanners exhibited considerable limitations. That being said, PCD-CT allowed for sharper image reconstructions with less pronounced blooming, which might be advantageous compared with EID-CT in the lumen range between 2 and 4 mm. This aspect should be investigated in future studies, especially in light of recent findings in cardiac PCD-CT^26–28^.

PCD-CT showed potential for considerable dose reduction compared to state-of-the-art EID-CT when aiming for the same image quality. With less than one third of the radiation exposure, PCD-CT images yielded the same SNR as the high-dose EID-CT protocol. This observation may be attributed to a higher intraluminal attenuation caused by the difference in detector-based weighting of low-energy photons. The higher attenuation in PCD-CT could aid in the differentiation of low-attenuating abutted material adjacent the stent wall, such as thrombus or intimal hyperplasia. The SNR advantage could also be used to reduce the required contrast media volume in patients with ASPAD, since these individuals commonly suffer from an impaired renal function. Both aspects are critical for patient safety and therefore desirable especially in the context of repeated examinations^29^.

Based on the results of our study, recommendations can be made for scan parameters and reconstruction settings in ASPAD patients. Superior image quality at reduced dose levels when using PCD-CT suggests its suitability for complex vascular assessments. Furthermore, our results indicate, that superior vascular assessment is feasible at a dose level far below the national diagnostic reference values of 7 mGy. One of the most significant advantages in PCD-CT lies in the increased spatial resolution when scanning in UHR mode, which allows for image reconstruction with sharper convolution kernels. While only used for the reference scan in the present study, UHR mode allows clinically significant improvements in small vessels or diameters^30^ and should be further investigated in the context of ASPAD. Especially in cases of stent malapposition or collapse, higher spatial resolution can facilitate a more accurate delineation of the individual stent struts^31,32^. Despite the lower SNR in comparison to smoother reconstructions, high spatial frequency has been deemed valuable in previous phantom^33^ and clinical analyses^23^. With smoother reformatting, the stent structure is not discernible and assessment of luminal contrast might be unreliable. This hinders the distinction between mild stent compression and complete stent collapse. Furthermore, the differentiation of luminal narrowing by intimal hyperplasia and real stent compression is essential to ascertain whether endovascular debulking strategies are promising.

Drawing from our findings, we recommend the use of low-dose PCD-CT and image reconstruction with the sharp BV76 kernel whenever scanning in standard-resolution mode. For EID-CT, we recommend using the sharpest kernel available (i.e., BV59) and high-dose scans within the legally permitted dose reference values to achieve adequate image quality. Furthermore, reconstructions with a small field-of-view separate for each leg help exploit the full resolution potential of the scanners. Obviously, PCD-CT results could be further improved when facilitating the UHR mode and sampling of sub-millimeter slice thickness but this was not in the scope of this study and discussions about the benefits and drawbacks of using UHR mode and very thin slices in daily practice must remain the topic of future studies.

Several limitations must be acknowledged when interpreting the study’s results. First, only one cadaver and a limited selection of stent types/vendors could be investigated. Second, the experimental setup did not allow to generate appositional stenosis inside the stents. This prohibited the assessment of abutted low attenuating material as encountered in partial thrombosis or intimal hyperplasia. Third, extensive vascular degeneration in the cadaver caused contrast leakage and subsequent extravasal contrast agent accumulation. Fourth, no scenarios with a residual luminal diameter between 2 mm and 4.5 mm could be examined. However, advantages for PCD-CT technology are conceivable, especially in situations with very little residual lumen. Fifth, ASPAD imaging is particularly challenging in below-the-knee arteries, which could not be studied with the presented model. Sixth, scan protocols were chosen for optimal comparability rather than optimising them to the respective scanner design. In particular, the benefits of PCD-CT regarding spatial resolution, the available Ultra-high resolution mode and dose-neutral spectral imaging might be underestimated^25^, which would push its limitations in very low luminial diameters even further. Seventh, the presented data are not sufficient to determine, wheter the found differences in objective image quality translate into clinical impact.

## Conclusion

This study confirms the results from previous analyses in disease-free vessels and gives recommendations for scan and reconstruction parameters suitable for ASPAD imaging. When choosing comparable reconstruction parameters, PCD-CT facilitates substantially higher SNR and luminal attenuation than EID-CT, indicating substantial radiation dose and contrast agent volume saving potential. PCD-CT scans allow reconstructions with sharper kernels and therefore higher spatial resolution, resulting in improved delineation of the stent lumen, wall, and structure as well as adjacent calcifications. This has notable implications for the eligibility for endovascular therapy and might impact the overall patient outcome.

## Supplementary Information

Supplementary Information.

## Data Availability

The datasets generated and/or analyzed during the current study are not publicly available but are available from the corresponding author on reasonable request. Due to the nature of this research, participants of this study did not agree for their data to be shared in a public repository.

## Abbreviations

ASPAD: Advanced stage peripheral arterial disease
CNR: Contrast-to-noise ratio
CTA: Computed tomography angiography
CTDI_vol_: Volume computed tomography dose index
EID-CT: Energy-integrating detector computed tomography
PAD: Peripheral arterial disease
PCD-CT: Photon-counting detector computed tomography
SNR: Signal-to-noise ratio
UHR: Ultra-high resolution
