# Supplementary material for: Diagnostic limitations in advanced stage peripheral arterial disease in a cadaveric study comparing photon-counting and energy-integrating CT detectors
Source: Sci Rep. 2025 Feb 26;15:6923. doi: 10.1038/s41598-025-91239-x (PMC11865436; doi:10.1038/s41598-025-91239-x)
Supplement: Supplementary file 1 — Supplementary Information. [file 41598_2025_91239_MOESM1_ESM.docx]

**Supplemental table 1: Lumen visibility and dose**

| **Protocol/ Dose** | 3 mGy | 5 mGy | 10 mGy |
| --- | --- | --- | --- |
| EID BV40 | 78,01 | 76,77 | 77,34 |
| EID BV49 | 81,24 | 82,03 | 81,95 |
| EID BV59 | 87,66 | 87,96 | 87,96 |
| PCD BV40 | 78,06 | 79,29 | 78,37 |
| PCD BV48 | 83,02 | 83,65 | 86,16 |
| PCD BV60 | 90,09 | 89,50 | 89,78 |
| PCD BV76 | 97,73 | 96,73 | 97,16 |

***Note.*** *Lumen visibility is given as mean %.*
